# Hantaviruses: a global disease problem.

**DOI:** 10.3201/eid0302.970202

**Published:** 1997

**Authors:** C Schmaljohn, B Hjelle

**Affiliations:** United States Army Medical Research Institute of Infectious Diseases, Fort Detrick, Frederick, Maryland 21702-5011, USA. cschmaljohn@detrick.army.mil

## Abstract

Hantaviruses are carried by numerous rodent species throughout the world. In 1993, a previously unknown group of hantaviruses emerged in the United States as the cause of an acute respiratory disease now termed hantavirus pulmonary syndrome (HPS). Before than, hantaviruses were known as the etiologic agents of hemorrhagic fever with renal syndrome, a disease that occurs almost entirely in the Eastern Hemisphere. Since the discovery of the HPS-causing hantaviruses, intense investigation of the ecology and epidemiology of hantaviruses has led to the discovery of many other novel hantaviruses. Their ubiquity and potential for causing severe human illness make these viruses an important public health concern; we reviewed the distribution, ecology, disease potential, and genetic spectrum.


### Hantaviruses: A Global Disease Problem

**Connie Schmaljohn* and Brian Hjelle†**
*United States Army Medical Research Institute of Infectious Diseases, Fort Detrick, Frederick, Maryland, USA; and
†University of New Mexico, Albuquerque, New Mexico, USA

Hantaviruses are carried by numerous rodent species throughout the world. In 1993, a previously unknown group of hantaviruses emerged in the United States as the cause of an acute respiratory disease now termed hantavirus pulmonary syndrome (HPS). Before then, hantaviruses were known as the etiologic agents of hemorrhagic fever with renal syndrome, a disease that occurs almost entirely in the Eastern Hemisphere. Since the discovery of the HPS-causing hantaviruses, intense investigation of the ecology and epidemiology of hantaviruses has led to the discovery of many other novel hantaviruses. Their ubiquity and potential for causing severe human illness make these viruses an important public health concern; we reviewed the distribution, ecology, disease potential, and genetic spectrum.

The genus *Hantavirus*, family Bunyaviridae, comprises at least 14 viruses, including those that cause hemorrhagic fever with renal syndrome (HFRS) and hantavirus pulmonary syndrome (HPS) (Table 1). Several tentative members of the genus are known, and others will surely emerge as their natural ecology is further explored. Hantaviruses are primarily rodent-borne, although other animal species har-boring hantaviruses have been reported. Unlike all other viruses in the family, hantaviruses are not transmitted by arthropod vectors but (most frequently) from inhalation of virus-contaminated aerosols of rodent excreta (1). Human-to-human transmission of hantaviruses has not been documented, except as noted below.

The recognition of a previously unknown group of hantaviruses as the cause of HPS in 1993 is an example of virus emergence due to environmental factors favoring of the natural reservoir; a larger reservoir increases opportunities for human infection. We reviewed the global distribution of hantaviruses, their potential to cause disease, and their relationships to each other and to their rodent hosts.

### History of HFRS and HPS

"Hemorrhagic fever with renal syndrome" denotes a group of clinically similar illnesses that occur throughout the Eurasian landmass and adjoining areas (2,3). HFRS includes diseases previously known as Korean hemorrhagic fever, epidemic hemorrhagic fever, and nephropathia epidemica (4). Although these diseases were recognized in Asia perhaps for centuries, HFRS first came to the attention of western physicians when approximately 3,200 cases occurred from 1951 to 1954 among United Nations forces in Korea (2,5). Other outbreaks of what is believed to have been HFRS were reported in Russia in 1913 and 1932, among Japanese troops in Manchuria in 1932 (2,6), and in Sweden in 1934 (7,8). In the early 1940s, a viral etiology for HFRS was suggested by Russian and Japanese investigators who injected persons with filtered urine or serum from patients with naturally acquired disease (2). These studies also provided the first clues to the natural reservoir of hantaviruses: the Japanese investigators claimed to produce disease in humans by injecting bacteria-free filtrates of tissues from *Apodemus agrarius* or mites that fed on the *Apodemus* mice. Mite transmission was never conclusively demonstrated by other investigators, and it was not until 1978 that a rodent reservoir for HFRS-causing viruses was confirmed by investigators who demonstrated that patient sera reacted with antigen in lung sections of wild-caught *Apodemus agrarius* and that the virus could be passed from rodent to rodent (9). The successful propagation of Hantaan (HTN) virus in cell culture in 1981 provided the first opportunity to study this pathogen systematically (10). The history of HFRS has been explored (2,11,12).

Address for correspondence: Connie Schmaljohn, Virology Division, USAMRID, Fort Detrick, Frederick, MD 21702-5011; fax: 301-619-2439; e-mail: cschmaljohn@detrick.army.mil

Table 1. Members of the genus *Hantavirus*, family Bunyaviridae

| Species | Disease | Principal Reservoir | Distribution of Virus | Distribution of Reservoir |
|---|---|---|---|---|
| Hantaan (HTN) | HFRS[a] | *Apodemus agrarius* (striped field mouse) | China, Russia, Korea | C Europe south to Thrace, Caucasus, & Tien Shan Mtns; Amur River through Korea to E Xizang & E Yunnan, W Sichuan, Fujiau, & Taiwan (China) |
| Dobrava-Belgrade (DOB) | HFRS | *Apodemus flavicollis* (yellow-neck mouse) | Balkans | England & Wales, from NW Spain, France, S Scandinavia through European Russia to Urals, S Italy, the Balkans, Syria, Lebanon, & Israel |
| Seoul (SEO) | HFRS | *Rattus norvegicus* (Norway rat) | Worldwide | Worldwide |
| Puumala (PUU) | HFRS | *Clethrionomys glareolus* (bank vole) | Europe, Russia, Scandinavia | W Palearctic from France and Scandinavia to Lake Baikai, south to N Spain, N Italy, Balkans,W Turkey, N Kazakhstan, Altai & Sayan Mtns; Britain & SW Ireland |
| Thailand (THAI) | nd[b] | *Bandicota indica* (bandicoot rat) | Thailand | Sri Lanka, peninsular India to Nepal, Burma, NE India, S China, Laos, Taiwan, Thailand, Vietnam |
| Prospect Hill (PH) | nd | *Microtus pennsylvanicus* (meadow vole) | U.S., Canada | C Alaska to Labrador, including Newfoundland & Prince Edward Island, Canada; Rocky Mountains to N New Mexico, in Great Plains to N Kansas, & in Appalachians to N Georgia, U.S. |
| Khabarovsk (KHB) | nd | *Microtus fortis* (reed vole) | Russia | Transbaikalia Amur region; E China |
| Thottapalayam (TPM) | nd | *Suncus murinus* (musk shrew) | India | Afghanistan, Pakistan, India, Sri Lanka, Nepal, Bhutan, Burma, China, Taiwan, Japan, Indomalayan Region |
| Tula (TUL) | nd | *Microtus arvalis* (European common vole) | Europe | Throughout Europe to Black Sea & NE to Kirov region, Russia |
| Sin Nombre (SN) | HPS[c] | *Peromyscus maniculatus* (deer mouse) | U.S., Canada, Mexico | Alaska Panhandle across N Canada, south through most of continental U.S., excluding SE & E seaboard, to southernmost Baja California Sur and to NC Oaxaca, Mexico |
| New York (NY) | HPS | *Peromyscus leucopus* (white-footed mouse) | U.S. | C and E U.S. to S Alberta & S Ontario, Quebec & Nova Scotia, Canada; to N Durango & along Caribbean coast to Isthmus of Tehuantepec & Yucatan Peninsula, Mexico |

[a]HFRS, hemorrhagic fever with renal syndrome
[b]nd, none documented
[c]HPS, hantavirus pulmonary syndrome

Table 1. Members of the genus *Hantavirus*, family Bunyaviridae (continued)

| Species | Disease | Principal Reservoir | Distribution of Virus | Distribution of Reservoir |
|---|---|---|---|---|
| Black Creek Canal (BCC) | HPS | *Sigmodon hispidus* (cotton rat) | U.S. | SE U.S., from S Nebraska to C Virginia south to SE Arizona & peninsular Florida; interior & E Mexico through Middle America to C Panama; in South America to N Colombia & N Venezuela |
| El Moro Canyon (ELMC)[d] | nd | *Reithrodontomys megalotis* (Western harvest mouse) | U.S., Mexico | British Columbia & SE Alberta, Canada; W and NC U.S., S to N Baja California & interior Mexico to central Oaxaca |
| Bayou (BAY)[d] | HPS | *Oryzomys palustris* (rice rat) | U.S. | SE Kansas to E Texas, eastward to S New Jersey & peninsular Florida |
| Probable species:[e] | | | | |
| Topografov (TOP) | nd | *Lemmus sibiricus* (Siberian lemming) | Siberia | Palearctic, from White Sea, W Russia, to Chukotski Peninsula, NE Siberia, & Kamchatka; Nearctic, from W Alaska E to Baffin Island & Hudson Bay, S Rocky Mtns to C B.C., Canada |
| Andes (AND)[d] | HPS | *Oligoryzomys longicaudatus*[f] (long-tailed pygmy rice rat) | Argentina | NC to S Andes, approximately to 50 deg S latitude, in Chile & Argentina |
| To be named[d] | HPS | *Calomys laucha* vesper mouse | Paraguay | N Argentina & Uruguay, SE Bolivia, W Paraguay, and WC Brazil |
| Isla Vista (ISLA)[d] | nd | *Microtus californicus* (California vole) | U.S. | Pacific coast, from SW Oregon through California, U.S., to N Baja California, Mexico |
| Bloodland Lake (BLL)[d] | nd | *Microtus ochrogaster* (prairie vole) | U.S. | N & C Great Plains, EC Alberta to S Manitoba, Canada, S to N Oklahoma & Arkansas, E to C Tennessee & W West Virginia, U.S.; relic populations elsewhere in U.S. & Mexico |
| Muleshoe (MUL)[d] | nd | *Sigmodon hipidus* (cotton rat) | U.S. | See Black Creek Canal |
| Rio Segundo (RIOS)[d] | nd | *Reithrodontomys mexicanus* (Mexican harvest mouse) | Costa Rica | S Tamaulipas & WC Michoacan, Mexico, S through Middle American highlands to W Panama; Andes of W Colombia & N Ecuador |
| Rio Mamore (RIOM)[d] | nd | *Oligoryzomys microtis* (small-eared pygmy rice rat) | Bolivia | C Brazil south of Rios Solimoes-Amazon & contiguous low lands of Peru, Bolivia, Paraguay, & Argentina. |

[d] not yet isolated in cell culture
[e] viruses for which incomplete characterization is available, but for which there is clear evidence indicating that they are unique
[f] suspected host, but not confirmed
Adapted from (57,72) and from (9,13,23,38,42,43,50-71)

HPS was first described in 1993 when a cluster of cases of adult fatal respiratory distress of unknown origin occurred in the Four Corners region of the United States (New Mexico, Arizona, Colorado, and Utah). The unexpected finding that sera from patients reacted with hantaviral antigens was quickly followed by the genetic identification of a novel hantavirus in patients' tissues and in rodents trapped near patients' homes (13-15).

### Prevalence and Clinical Course

Approximately 150,000 to 200,000 cases of HFRS involving hospitalization are reported each year throughout the world, with more than half in China (16). Russia and Korea also report hundreds to thousands of HFRS cases each year. Most remaining cases (hundreds per year) are found in Japan, Finland, Sweden, Bulgaria, Greece, Hungary, France, and the Balkan countries formerly constituting Yugoslavia (16). Depending in part on which hantavirus is responsible for the illness, HFRS can appear as a mild, moderate, or severe disease (Table 2). Death rates range from less than 0.1% for HFRS caused by Puumala (PUU) virus to approximately 5% to 10% for HFRS caused by HTN virus (16). The clinical course of severe HFRS involves five overlapping stages: febrile, hypotensive, oliguric, diuretic, and convalescent; it is not uncommon, however, for one or more of these stages to be inapparent or absent. The onset of the disease is usually sudden with intense headache, backache, fever, and chills. Hemorrhage, if it occurs, is manifested during the febrile phase as a flushing of the face or injection of the conjunctiva and mucous membranes. A petechial rash may also appear, commonly on the palate and axillary skin folds. Sudden and extreme albuminuria, around day 4, is characteristic of severe HFRS. As the febrile stage ends, hypotension can abruptly develop and last for hours or days, during which nausea and vomiting are common. One-third of deaths occur during this phase because of vascular leakage and acute shock. Almost half of all deaths occur during the subsequent (oliguric) phase because of hypervolemia. Patients who survive and progress to the diuretic phase show improved renal function but may still die of shock or pulmonary complications. The final (convalescent) phase can last weeks to months before recovery is complete (3,5,12).

More than 250 cases of HPS have been reported throughout North and South America. Although the disease has many features (e.g., a febrile prodrome, thrombocytopenia, and leukocytosis) in common with HFRS (Table 2), in HPS capillary leakage is localized exclusively in the lungs, rather than in the retroperitoneal space, and the kidneys are largely unaffected. Most of the 174 cases of HPS in the United States and Canada have been caused by Sin Nombre (SN) virus. In HPS, death occurs from shock and cardiac complications, even with adequate tissue

Table 2. Distinguishing clinical characteristics for HFRS and HPS

| Disease | Pathogens | Distinguishing Characteristics* |
|---|---|---|
| HFRS (moderate-severe)<br>Death rate<br>1%-15% | HTN, SEO, DOB | hemorrhage +++<br>azotemia/<br>proteinuria +++/++++<br>pulmonary capillary leak +/++<br>myositis +/+++<br>conjunctival injection ++/++++<br>eye pain/myopia ++/++++ |
| HFRS (mild)<br>Death rate <1% | PUU | hemorrhage +<br>azotemia/<br>proteinuria +/++++<br>pulmonary capillary leak -/+<br>myositis +<br>conjunctival injection +<br>eye pain/myopia ++/++++ |
| HPS (prototype)<br>Death rate >40% | SN, NY | hemorrhage +<br>azotemia/<br>proteinuria +<br>pulmonary capillary leak ++++<br>myositis -<br>conjunctival injection -/+<br>eye pain/myopia - |
| HPS (renal variant)<br>Death rate>40% | BAY, BCC, Andes | hemorrhage +<br>azotemia/<br>proteinuria ++/+++<br>pulmonary capillary leak +++/++++<br>myositis ++/++++<br>conjunctival injection -/++<br>eye pain/myopia - |

*Minimum/maximum occurrence of the characteristic: - rarely reported; + infrequent or mild manifestation; ++, +++, ++++ more frequent and severe manifestation.

oxygenation. Cases of HPS in the southeastern United States, as well as many in South America, are caused by a newly recognized clade (a group that shares a common ancestor) of viruses that includes Bayou (BAY), Black Creek Canal (BCC), and Andes viruses. As with HFRS, clinical differences can be observed among patients with HPS caused by different hantaviruses. For example, although HPS due to SN virus infection can sometimes be associated with renal insufficiency after prolonged hypoperfusion, renal impairment is only rarely observed early in disease, and chemical evidence of skeletal muscle inflammation (increased serum levels of the muscle enzyme creatine kinase) is rare (17). In contrast, both renal insufficiency and elevated creatine kinase levels are observed at much higher frequency, although not universally, with Andes, BAY, and BCC virus infections (18-20; J. Davis, J. Cortes, and C. Barclay, pers. comm.). In an outbreak of HPS recently described in Paraguay, a novel hantavirus, carried by *Calomys laucha*, was identified as the etiologic agent (21). The relationship of this virus to other HPS-causing hantaviruses remains to be established.

### Ecology and Epidemiology

Hantavirus infection is apparently not deleterious to its rodent reservoir host and is associated with a brisk antibody response against the virion envelope and core proteins and chronic, probably lifelong infection. In natural populations, most infections occur through age-dependent horizontal route(s). The highest antibody prevalence is observed in large (mature) animals. A striking male predilection for hantavirus infection is observed in some rodent species such as harvest mice and deer mice, but not in urban rats (*Rattus norvegicus*) (22-24). Horizontal transmission among cage-mates was experimentally demonstrated (25), but vertical transmission from dam to pup is negligible or absent both in wild and experimental settings (22,24,25).

Outbreaks of hantaviral disease have been associated with changes in rodent population densities, which may vary greatly across time, both seasonally and from year to year. Cycles respond to such extrinsic factors as interspecific competition, climatic changes, and predation. Spring and summer outbreaks of HFRS in agricultural settings in Asia and Europe are linked to human contact with field rodents through the planting and harvesting of crops (16,26). PUU outbreaks in Scandinavia and the HPS outbreak in the Four Corners region of the United States were associated with natural rodent population increases, followed by invasion of buildings by rodents (27,28). The ecologic events that led to 1994 and 1996 outbreaks of Andes virus-HPS in Patagonia, a region in southern South America, are being investigated. Human interventions, such as the introduction of Old World plant species (e.g., rosas mosquetas and Scottish brougham) to Patagonia, with associated alteration in rodent population dynamics, have been suggested as possible factors. Recent fires and a mild winter in Argentina's Rio Negro and Chubut Provinces may also have had a positive effect on the carrier rodent, the colilargo, *Oligoryzomys longicaudatus* (M. Christie and O. Pearson, pers. comm.).

Although the aerosol route of infection is undoubtedly the most common means of transmission among rodents and to humans, virus transmission by bite may occur among certain rodents (29) and may also occasionally result in human infection (30) (often inside a closed space, such as a rodent-infested grain silo, garage, or outbuilding used for food storage). Epidemiologic investigations have linked virus exposure to such activities as heavy farm work, threshing, sleeping on the ground, and military exercises. Indoor exposure was linked to invasion of homes by field rodents during cold weather or to nesting of rodents in or near dwellings (16,31). Genetic sequencing of rodent- and patient-associated viruses has been used to pinpoint the precise locations of human infections, which has supported the role of indoor exposure in hantavirus transmission (32,33). Many hantavirus infections have occurred in persons of lower socioeconomic status because poorer housing conditions and agricultural activities favor closer contact between humans and rodents. However, suburbanization, wilderness camping, and other outdoor recreational activities have spread infection to persons of middle and upper incomes.

Nosocomial transmission of hantaviruses has not been documented until very recently (34) and must be regarded as rare. However, viruses have been isolated from blood and urine of HFRS patients, so exposure to bodily fluids of infected persons could result in secondary transmission. Only rarely have multiple North American HPS cases been associated with particular households or buildings. During recent outbreaks of HPS in South America, however, clustering of cases in households and among personal contacts appeared to be more common (M. Christie, pers. comm.). During a recent outbreak of Andes-virus–associated HPS in Patagonia, a Buenos Aires physician apparently contracted the infection after minimal exposure to infected patient blood (34; D.A. Pirola, pers. comm.). An adolescent patient in Buenos Aires apparently contracted hantavirus infection from her parents, who were infected in Patagonia. This unprecedented observation of apparent person-to-person spread of a hantavirus clearly requires laboratory confirmation, especially by careful comparative analysis of the viral sequences (32,33).

Hantaviruses have also caused several laboratory-associated outbreaks of HFRS. Laboratory-acquired infections were traced to persistently infected rats obtained from breeders (35-37), to wild-caught, naturally infected rodents (38-40), or to experimentally infected rodents (39). No illnesses due to laboratory infections have been reported among workers using cell-culture adapted viruses, although asymptomatic seroconversions have been documented (40).

### Hantavirus Distribution and Disease-causing Potential

The worldwide distribution of rodents known to harbor hantaviruses (Table 1) suggests great disease-causing potential. Each hantavirus appears to have a single predominant natural reservoir. With rare exception, the phylogenetic interrelationships among the viruses and those of their predominant host show remarkable concordance (Figure; 41). These observations suggest that hantaviruses do not adapt readily to new hosts and that they are closely adapted for success in their host, possibly because of thousands of years of coexistence. As many as three hantaviruses can be found in a particular geographic site, each circulating in its own rodent reservoir, with no apparent evolutionary influence on one another (42).

All known hantaviruses, except Thottapalayam (TPM) virus, have been isolated or detected in murid rodents. Because only one isolate of TPM virus was made from a shrew (Order Insectivora), it is not clear if *Suncus* is the true primary reservoir or an example of a "spillover" host, i.e., a secondary host infected through contact with the primary host. Spillover is common in sympatric murid rodents, including those identified as the predominant carrier of another hantavirus; thus, the opportunity for genetic exchange among hantaviruses is present in nature. Spillover hosts are believed to have little or no impact on hantaviral distribution or associated disease. However, rodents other than the primary reservoirs can play an important carrier role. For example, *Microtus rossiaemeridionalis* may play a role in maintenance of Tula virus in some settings (43), and *Peromyscus leucopus* and *Peromyscus boylii* can be important reservoirs for SN virus in the western United States (T. Yates and B. Hjelle, unpub. data). Apparent spillover may also be the result of laboratory errors such as polymerase chain reaction (PCR) contamination or misidentification of rodent species. However, spillover is probably underappreciated in many studies that rely on reverse transcriptasePCR for identifying specific viruses because many primer pairs may not detect an unexpected spillover virus. In either case, because mistaken identities and cell culture contaminations with other hantaviruses have occasionally been reported, investigators should verify unusual findings to prevent further confusion.

### Antigenic and Genetic Diversity among Hantaviruses

Hantaviruses have been characterized by a combination of antigenic and genetic methods. For viruses propagated in cell culture, the plaque-reduction neutralization test is the most sensitive serologic assay for differentiation (44,45); nine hantaviruses have been defined by this test: HTN, Seoul (SEO), PUU, Prospect Hill, Dobrava-Belgrade (DOB), Thailand, TPM, SN, and BCC viruses (44-48). Genetic relationships among hantaviruses are mirrored in their antigenic properties. A direct correlation between genetic and antigenic relationships is difficult; however, it can be assumed that the plaque-reduction neutralization test measures differences in the M segment gene products, i.e., the G1 and G2 envelope glycoproteins. Comparing the deduced G1 and G2 amino acid sequences, therefore, may provide clues to the antigenic as well as genetic diversity among hantaviruses.

Of characterized hantavirus isolates, SEO virus is the most genetically homogeneous. Isolates of SEO virus, regardless of their geographic origin, display M segment nucleotide and deduced amino acid sequence homologies of approximately 95%, and 99%, respectively (41,47). A reported exception, the R22 isolate from China, had a slightly lower homology;

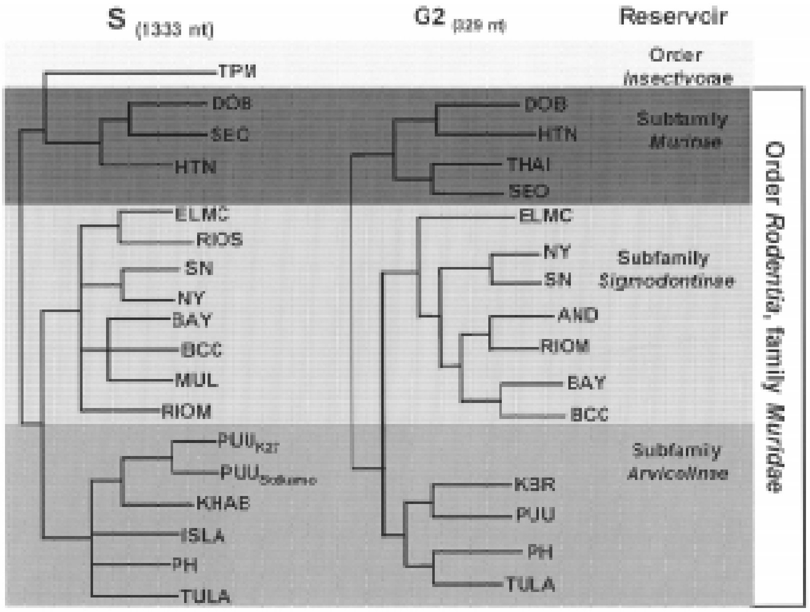

Figure. Phylogeny of hantaviruses and their relationships to natural reservoirs. The trees were constructed by comparing the complete coding regions of the S segments of hantaviruses or of 330 nucleotides corresponding to those of the M segment of Hantaan virus (strain 76118) from nucleotides 1987 to 2315. Abrreviations for viruses are as in Table 1. For each analysis, a single most parsimonious tree was derived by using PAUP 3.1.1 software. For the S segment tree, boostrap values resulting from 100 replications were all greater than 87% except for the branch leading to BCC (78%) and the branch leading to DOB (52%). The next most common placing of DOB was on a branch with HTN.

however, the original data suggest that an error in the nucleotide sequence, resulting in a frame shift reading error, may account for almost all of the additional changes. PUU virus isolates vary the most, with M segment nucleotide and amino acid sequence homologies of 83% and 94% observed between a Finnish and Russian isolate. HTN virus also appears to be quite stable in nature. Comparing the M segment sequences of prototype HTN virus (from *Apodemus*) and those of two human isolates obtained at a 6-year interval from HFRS patients in Korea produced nucleotide and deduced amino acid sequence homologies of 94% and 97%, respectively (48). For SN virus, comparing the complete M or S segment sequences of three strains from California or New Mexico produced homologies of 87% to 99%. Partial nucleotide sequence comparisons of the M or S segments of SN viruses from adjacent counties in California, detected in deer mice captured 19 years apart, were 97.5% homologous (49). Similarly, a retrospective analysis of archived tissue samples collected in Mono County, California, in 1983 showed viruses with partial M and S segment nucleotide sequence homologies of about 87% with SN from an 1993 HPS patient from New Mexico (50). In all cases, the amino acid sequences encoded by these genes differed between cognate proteins by much less than 5%. These values are similar to those observed among strains of HTN virus. Studies have just begun to appear in which the nature of quasispecies in natural rodent hosts is defined (43,51). Such investigations should provide more definitive data concerning the genetic diversity among hantaviruses in nature.

### Evolution of Hantaviruses

Phylogenetic trees derived by comparing complete or partial S (Figure), M, or L segment nucleotide sequences (41,52,53) show two major lineages of hantaviruses, one leading to HTN, SEO, Thailand, and DOB viruses, and one leading to PUU, Prospect Hill, SN, and other New World hantaviruses. TPM virus, the first hantavirus isolated in cell culture (54), may be the most antigenically and genetically disparate member of the genus; however, comparison of the complete nucleotide sequence of the TPM S segment (A. Toney, B. Meyer, C. Schmaljohn, unpub. data) suggests that TPM virus is more closely related to HTN, SEO, and DOB viruses than to any of the other viruses in the genus (Figure). Nucleotide sequence homologies of the M and S segments of any two hantaviruses have approximately the same degree of divergence between each of the three segments, which suggests similar evolutionary rates for these two gene segments. A slightly higher homology among L segments sequenced to date perhaps indicates a greater need for conservation of either RNA or protein functions (47). Point mutations appear to account for most of the genetic drift among hantaviruses. Recombination has not been reported for hantaviruses, although segment reassortment within a particular species appears common (52,55). The exchange of gene segments is suggested to be nonrandom, with a higher propensity for M segment swapping, than for S or L (55). Whether it contributes to the pathogenesis of hantaviruses is not known, but reassortment certainly provides an avenue for more rapid accumulation of changes than could occur by point mutation. There is no evidence that reassortment can occur between different species of hantaviruses; however, this has not been studied systematically.

Murid rodents have probably harbored inapparent hantavirus infections for thousands, perhaps millions of years. It is likely that the genus *Hantavirus* evolved in the Old World and that viruses were carried by rodents across the Bering land bridge when they migrated during the Oligocene, and into South America in the Pliocene (71). Humans are incidental hosts, the victims of spillover infections from the natural host rodents. One of the two major forms of hanta-viral diseases is endemic in each hemisphere. Both HFRS and HPS can be divided into distinct clinical subtypes, and the viral strain is a key determinant of the severity and nature of the clinical abnormalities. Not covered in this review are clinical studies of HFRS and HPS patients, which suggest that pathogenesis may be immunologic and may be mediated by cytokine responses (72). New outbreaks with novel hantavirus strains are still being uncovered, especially in South America. However, the largest clinical caseload and largest number of deaths occur in Asia and Europe.

Dr. Schmaljohn is chief, Department of Molecular Virology, USAMRID. Current research interests include the development of molecular vaccines for hantaviruses, filoviruses, and flaviviruses.

Dr. Hjelle has been active in studies of the molecular biology, evolution, epidemiology, and clinical aspects of hantavirus disease. His laboratory is a reference diagnostic center for hantavirus infections of humans and animals and has recently received funding to develop innovative vaccine strategies against HPS and other emerging viral diseases.

### References

1. Lee H, van der Groen G. Hemorrhagic fever with renal syndrome. Prog Med Virol 1989;36:62-102.
2. Gajdusek D. Virus hemorrhagic fevers. J Pediatr 1962;60:841-57.
3. World Health Organization. Haemorrhagic fever with renal syndrome: memorandum from a WHO meeting. Bull World Health Organ 1983;61:269-75.
4. Gajdusek DC, Goldfarb LG, Goldgaber D. Bibliography of hemorrhagic fever with renal syndrome. Second Edition. Bethesda (MD): National Institutes of Health; 1987 Pub No. 88-3603.
5. Smadel J. Epidemic hemorrhagic fever. Am J Public Health 1953;43:1327-30.
6. Casals J, Henderson BE, Hoogstraakm G. A review of Soviet viral hemorrhagic fevers. J Infect Dis 1969;122:437-53.
7. Zetterholm SG. Akuta nefriter simulerande akuta bukfall. Svenska Lakartidningen 1934;31.
8. Myhrman G. En njursjukdom med egenartad symptombild. Nord Med Tidskr 1934;7:793-4.
9. Lee H, Lee P, Johnson K. Isolation of the etiologic agent of Korean hemorrhagic fever. J Infect Dis 1978;137:298-308.
10. French G, Foulke R, Brand O, Eddy G. Korean hemorrhagic fever: propagation of the etiologic agent in a cell line of human origin. Science 1981;211:1046-8.
11. Traub R. Korean hemorrhagic fever. J Infect Dis 1978;138:267-72.
12. McKee K, LeDuc J, Peters C. Hantaviruses. In: Belshe RB, Belshe RB, editors. Textbook of human virology. St Louis: Mosby Year Book 1991:615-32.
13. Nichol ST, Spiropoulou CF, Morzunov S, Rollin PE, Ksiazek TG, Feldmann H, et al. Genetic identification of a hantavirus associated with an outbreak of acute respiratory illness. Science 1993;262:914-7.
14. Ksiazek TG, Peters CJ, Rollin PE, Zaki S, Nichol S, Spiropoulou C, et al. Identification of a new North American hantavirus that causes acute pulmonary insufficiency. Am J Trop Med Hyg 1995;52:117-23.
15. Schmaljohn AL, Li D, Negley DL, Bressler DS, Turell MJ, Korch GW, et al. Isolation and initial characterization of a newfound hantavirus from California. Virology 1995;206:963-72.
16. Lee HW. Epidemiology and pathogenesis of hemorrhagic fever with renal syndrome. In: Elliott RM, editor. The Bunyaviridae. New York: Plenum Press, 1996:253-67.
17. Duchin JS, Koster FT, Peters CJ, Simpson GL, Tempest B, Zaki SR, et al. Hantavirus pulmonary syndrome: a clinical description of 17 patients with a newly recognized disease. N Engl J Med 1994;330:949-55.

18. Khan AS, Spiropoulou CF, Morzunov S, Zaki SR, Kohn MA, Nawas SR, et al. Fatal illness associated with a new hantavirus in Louisiana. J Med Virol 1995;46:281-6.
19. Khan AS, Gaviria JM, Rollin PE, Hlady WG, Ksiazek TG, Armstrong LR, et al. Hantavirus pulmonary syndrome in Florida: association with the newly identified Black Creek Canal virus. Am J Med 1996;100:46-8.
20. Hjelle B, Goade D, Torrez-Martínez N, Lang-Williams M, Kim J, Harris RL, et al. Hantavirus pulmonary syndrome, renal insufficiency and myositis associated with infection by Bayou hantavirus. Clin Infect Dis 1996;23:495-500.
21. Williams R, Bryan R, Mills H, Palma I, Vera I, Velasquez F, et al. Hantavirus pulmonary syndrome in western Paraguay. Am J Trop Med Hyg 1996;55 (suppl), Abstract 30.
22. Mills J, Ksiazek T, Ellis B, Rollin P, Nichol S, Yates T, et al. Patterns of association with host and habitat: antibody reactivity with Sin Nombre virus in small mammals in the major biotic communities of the southwestern United States. Am J Trop Med Hyg. In press.
23. Hjelle B, Anderson B, Torrez-Martínez N, Song W, Gannon WF, L. YT. Prevalence and geographic genetic variation of hantaviruses of New World harvest mice (*Reithrodontomys*): identification of a divergent genotype from a Costa Rican *Reithrodontomys mexicanus*. Virology 1995;207:452-9.
24. Childs J, Korch G, Glass G, LeDuc J, Shah K. Epizootiology of hantavirus infections in Baltimore: isolation of a virus from Norway rats and characteristics of infected rat populations. Am J Epidemiol 1987;126:55-68.
25. Lee H, Lee P, Baek L, Song C, Seong I. Intraspecific transmission of Hantaan virus, etiologic agent of Korean hemorrhagic fever, in the rodent *Apodemus agrarius*. Am J Trop Med Hyg 1981;30:1106-12.
26. Chen H-X, Qiu F-X, Dong B-J, Ji S-Z, Li Y-T, Wang Y, et al. Epidemiologic studies on hemorrhagic fever with renal syndrome in China. J Infect Dis 1986;154:394-8.
27. Niklasson B, LeDuc J. Epidemiology of nephropathia epidemica in Sweden. J Infect Dis 1987;155:269-76.
28. Parmenter R, Vigil R. The hantavirus epidemic in the southwest: an assessment of autumn rodent densities and population demographics in central and northern New Mexico. Atlanta (GA): 1993 Report to the Federal Centers for Disease Control and Prevention.
29. Glass G, Childs J, Korch G, LeDuc J. Association of intraspecific wounding with hantaviral infection in wild rats (*Rattus norvegicus*). Epidemiol Infect 1988;101:459-72.
30. Dournon E, Moriniere B, Matheron S, Girard P, Gonzalez J, Hirsch F, et al. HFRS after a wild rodent bite in the hautesavoie--and risk of exposure to Hantaan-like virus in Paris laboratory. Lancet 1984;1:676-7.
31. Armstrong L, Zaki S, Goldoft M, Todd R, Khan A, Khabbaz R, et al. Hantavirus pulmonary syndrome associated with entering or cleaning rarely used, rodent-infested structures. J Infect Dis 1995;172:1166.
32. Hjelle B, Torrez-Martínez N, Koster FT, Jay M, Ascher MS, Brown T, et al. Epidemiologic linkage of rodent and human hantavirus genomic sequences in case investigations of hantavirus pulmonary syndrome. J Infect Dis 1996;173:781-6.
33. Jay M, Hjelle B, Davis R, Ascher M, Baylies HN, Reilly K, et al. Occupational exposure leading to hantavirus pulmonary syndrome in a utility company employee. Clin Infect Dis 1996;22:841-4
34. Enria D, Padula P, Segura EL, Pini N, Edelstein A, Riva Posse C, et al. Hantavirus pulmonary syndrome in Argentina: possibility of person to person transmission. Medicina (B. Aires) 1996;56:709-11.
35. Umenai T, Lee P, Toyoda T, Yoshinaga K, Lee H, Saito T, et al. Korean hemorrhagic fever in staff in an animal laboratory. Lancet 1979;1:1314-6.
36. Desmyter J, LeDuc J, Johnson K, Brasseur F, Deckers C, Van Ypersele de Strihou C. Laboratory rat associated outbreak of haemorrhagic fever with renal syndrome due to Hantaan-like virus in Belgium. Lancet 1983;2:1445-8.
37. Lloyd G, Bowen E, Jones N. HFRS outbreak associated with laboratory rats in UK. Lancet 1984;May:1775-6.
38. Brummer-Korvenkontio M, Vaheri A, von Bonsdorff C-H, Vuorimies J, Manni T, Penttinen K, et al. Nephropathia epidemica: detection of antigen in bank voles and serologic diagnosis of human infection. J Infect Dis 1980;141:131-4.
39. Lee H, Johnson K. Laboratory-acquired infections with hantaan virus, the etiologic agent of Korean hemorrhagic fever. J Infect Dis 1982;146:645-51.
40. Centers for Disease Control and Prevention. Laboratory management of agents associated with hantavirus pulmonary syndrome: interim biosafety guidelines. MMWR Morb Mortal Wkly Rept 1994; 43:RR-7,1-2.
41. Xiao SY, Leduc JW, Chu YK, Schmaljohn CS. Phylogenetic analyses of virus isolates in the genus *Hantavirus*, family Bunyaviridae. Virology 1994;198:205-17.
42. Rawlings J, Torrez-Martínez N, Neill S, Moore G, Hicks B, Pichuantes S, et al. Cocirculation of multiple hantaviruses in Texas, with characterization of the S genome of a previously-undescribed virus of cotton rats (*Sigmodon hispidus*). Am J Trop Med Hyg 1996;55:672-9.
43. Plyusnin A, Vapalahti O, Lankinen H, Lehvaslaiho H, Apekina N, Myasnikov Y, et al. Tula virus: a newly detected hantavirus carried by European common voles. J Virol 1994;68:7833-9.
44. Schmaljohn C, Hasty S, Dalrymple J, LeDuc J, Lee H, von Bonsdorff C-H, et al. Antigenic and genetic properties of viruses linked to hemorrhagic fever with renal syndrome. Science 1985;227:1041-4.
45. Chu Y-K, Rossi C, LeDuc J, Lee H, Schmaljohn C, Dalrymple J. Serological relationships among viruses in the Hantavirus genus, family Bunyaviridae. Virology 1994;198:196-204.
46. Chu Y-K, Jennings G, Schmaljohn A, Elgh F, Hjelle B, Lee HW, et al. Cross-neutralization of hantaviruses with immune sera from experimentally-infected animals and from hemorrhagic fever with renal syndrome and hantavirus pulmonary syndrome patients. J Infect Dis 1995;172:1581-4.
47. Schmaljohn CS. Molecular Biology of Hantaviruses. In: Elliott RM, editor. The Bunyaviridae. New York: Plenum Press, 1996:63-90.

48. Schmaljohn C, Arikawa J, Hasty S, Rasmussen L, Lee WH, Lee PW, Dalrymple J. Conservation of antigenic properties and sequences encoding the envelope proteins of prototype hantaan virus and two virus isolates from Korean haemorrhagic fever patients. J Gen Virol 1988;69:1949-55.
49. Jay M, Ascher MS, Chomel BB, Madon M, Sesline D, Enge B, et al. Sero-epidemiological studies of hantavirus infection among wild rodents in California. Emerg Infect Dis 1997;3.
50. Nerurkar VR, Song JW, Song KJ, Nagle JW, Hjelle B, Jenison S, et al. Genetic evidence for a hantavirus enzootic in deer mice (*Peromyscus maniculatus*) captured a decade before the recognition of hantavirus pulmonary syndrome. Virology 1994;204:563-8.
51. Rowe JE, St Jeor SC, Riolo J, Otteson EW, Monroe MC, Henderson WW, et al. Coexistence of several novel hantaviruses in rodents indigenous to North America. Virology 1995;213:122-30.
52. Li D, Schmaljohn A, Anderson K, Schmaljohn C. Complete nucleotide sequences of the M and S segments of two hantavirus isolates from California: evidence for reassortment in nature among viruses related to hantavirus pulmonary syndrome. Virology 1995;206:973-83.
53. Spiropoulou CF, Morzunov S, Feldmann H, Sanchez A, Peters CJ, Nichol ST. Genome structure and variability of a virus causing hantavirus pulmonary syndrome. Virology 1994;200:715-23.
54. Carey D, Reuben R, Panicker K, Shope R, Myers R. Thottapalayam virus: a presumptive arbovirus isolated from a shrew in India. Indian J Med Res 1971;59:1758-60.
55. Henderson WW, Monroe MC, St Jeor SC, Thayer WP, Rowe JE, Peters CJ, et al. Naturally occurring Sin Nombre virus genetic reassortants. Virology 1995;214:602-10.
56. Hjelle B, Jenison S, Torrez-Martínez N, Yamada T, Nolte K, Zumwalt R, et al. A novel hantavirus associated with an outbreak of fatal respiratory disease in the southwestern United States: evolutionary relationships to known hantaviruses. J Virol 1994;68:592-6.
57. Hjelle B, Jenison S, Goade D, Green W, Feddersen R, Scott A. Hantaviruses: clinical, microbiologic, and epidemiologic aspects. Crit Rev Clin Lab Sci 1995;32:469-508.
58. Avsic-Zupanc T, Xiao SY, Stojanovic R, Gligic A, van der Groen G, LeDuc JW. Characterization of Dobrava virus: a Hantavirus from Slovenia, Yugoslavia. J Med Virol 1992;38:132-7.
59. Lee H, Baek L, Johnson K. Isolation of hantaan virus, the etiologic agent of Korean hemorrhagic fever from wild urban rats. J Infect Dis 1982;146:638-44.
60. Elwell M, Ward G, Tingpalapong M, LeDuc J. Serologic evidence of hantaan-like virus in rodents and man in Thailand. Southeast Asian J Trop Med Public Health 1985;16:349-54.
61. Lee P, Amyx H, Yanagihara R, Gajdusek D, Goldgaber D, Gibbs C Jr. Partial characterization of Prospect Hill virus isolated from meadow voles in the United States. J Infect Dis 1985;152:826-9.
62. Hörling J, Chizhikov V, Lundkvist Å, Jonsson M, Ivanov L, Dekonenko A, et al. Khabarovsk virus: a phylogenetically and serologically distinct hantavirus isolated from *Microtus fortis* trapped in far-east Russia. J Gen Virol 1996;77:687-94.
63. Elliott LH, Ksiazek TG, Rollin PE, Spiropoulou CF, Morzunov S, Monroe M, et al. Isolation of the causative agent of hantavirus pulmonary syndrome. Am J Trop Med Hyg 1994;51:102-8.
64. Song J-W, Baek L-J, Gajdusek DC, Yanagihara R, Gavrilovskaya I, Luft BJ, et al. Isolation of pathogenic hantavirus from white footed mouse (*Peromyscus leucopus*). Lancet 1994;344:1637.
65. Ravkov EV, Rollin PE, Ksiazek TG, Peters CJ, Nichol ST. Genetic and serologic analysis of Black Creek Canal virus and its association with human disease and *Sigmodon hispidus* infection. Virology 1995;210:482-9.
66. Torrez-Martínez N, Song W, Hjelle B. Nucleotide sequence analysis of the M genomic segment of El Moro Canyon hantavirus: antigenic distinction from Four Corners hantavirus. Virology 1995;211:336-8.
67. Morzunov SP, Feldmann H, Spiropoulou CF, Semenova VA, Rollin PE, Ksiazek TG, et al. A newly recognized virus associated with a fatal case of hantavirus pulmonary syndrome in Louisiana. J Virol 1995;69:1980-3.
68. Plyusnin A, Vapalahti O, Lundkvist A, Henttonen H, Vaheri A. Newly recognised hantavirus in Siberian lemmings [letter]. Lancet 1996;347:1835.
69. López N, Padula P, Rossi C, Lázaro ME, Franze-Fernández MT. Genetic identification of a new hantavirus causing severe pulmonary syndrome in Argentina. Virology 1996;220:223-6.
70. Song W, Torrez-Martínez N, Irwin W, Harrison J, Davis R, Ascher M, et al. Isla Vista virus: a genetically novel hantavirus of the California vole *Microtus californicus*. J Gen Virol 1995;76:3195-9.
71. Hjelle B, Torrez-Martínez N, Koster FT. Hantavirus pulmonary syndrome-related virus from Bolivia. Lancet 1996;347:57.
72. Mertz G, Hjelle B, Bryan R. Hantavirus infection. In: Fauci A, Schrier R, editors. Advances in internal medicine. Chicago: Mosby Year Book Inc., 1996:373-425.
